# Microalgae: A Promising Source of Valuable Bioproducts

**DOI:** 10.3390/biom10081153

**Published:** 2020-08-06

**Authors:** Vyacheslav Dolganyuk, Daria Belova, Olga Babich, Alexander Prosekov, Svetlana Ivanova, Dmitry Katserov, Nikolai Patyukov, Stanislav Sukhikh

**Affiliations:** 1 Institute of Living Systems, Immanuel Kant Baltic Federal University, A. Nevskogo Street 14, 236016 Kaliningrad, Russia; dolganuk_vf@mail.ru (V.D.); antonina-daria@mail.ru (D.B.); olich.43@mail.ru (O.B.); dkatze39@gmail.com (D.K.); sved_08_89@mail.ru (N.P.); stas-asp@mail.ru (S.S.); 2 Laboratory of Biocatalysis, Kemerovo State University, Krasnaya Street 6, 650043 Kemerovo, Russia; a.prosekov@inbox.ru; 3 Natural Nutraceutical Biotesting Laboratory, Kemerovo State University, Krasnaya Street 6, 650043 Kemerovo, Russia; 4 Department of General Mathematics and Informatics, Kemerovo State University, Krasnaya Street 6, 650043 Kemerovo, Russia

**Keywords:** microalgae, biologically active substances, proteins, lipids, polysaccharides, vitamins, pigments

## Abstract

Microalgae are a group of autotrophic microorganisms that live in marine, freshwater and soil ecosystems and produce organic substances in the process of photosynthesis. Due to their high metabolic flexibility, adaptation to various cultivation conditions as well as the possibility of rapid growth, the number of studies on their use as a source of biologically valuable products is growing rapidly. Currently, integrated technologies for the cultivation of microalgae aiming to isolate various biologically active substances from biomass to increase the profitability of algae production are being sought. To implement this kind of development, the high productivity of industrial cultivation systems must be accompanied by the ability to control the biosynthesis of biologically valuable compounds in conditions of intensive culture growth. The review considers the main factors (temperature, pH, component composition, etc.) that affect the biomass growth process and the biologically active substance synthesis in microalgae. The advantages and disadvantages of existing cultivation methods are outlined. An analysis of various methods for the isolation and overproduction of the main biologically active substances of microalgae (proteins, lipids, polysaccharides, pigments and vitamins) is presented and new technologies and approaches aimed at using microalgae as promising ingredients in value-added products are considered.

## 1. Introduction

Recently, the issues of microalgae cultivation have been of increasing interest among researchers due to their ability to synthesize various biologically active substances, the rapid growth of biomass and the ability to adjust their biochemical composition depending on cultivation conditions. Microalgae are marine or freshwater microorganisms consisting of a single eukaryotic cell. These are unicellular flora representatives with huge potential for application in various branches of science and technology [1]. Previously, cyanobacteria, which later became considered bacteria, were also classified as blue-green algae. Currently, there are many types of eukaryotic unicellular microorganisms. Their diversity can be compared with the diversity of insects [1,2]. Unlike heterotrophic microorganisms, which require various organic compounds for growth, unicellular photosynthetic organisms produce biomass from completely oxidized inorganic substances and mineral elements due to the light energy converted during photosynthesis. Furthermore, microalgae biomass production technologies do not pollute the environment, use carbon dioxide while generating oxygen, consume a relatively small amount of water and may occupy land unsuitable for cultivation of agricultural crops [2].

At present, two main areas of use of microalgae can be distinguished: the production of biomass as a biologically active additive, and the cultivation of microalgae for the subsequent isolation of biologically active substances from biomass.

Microalgae are rich in nutrients and biologically active substances, such as proteins, polysaccharides, lipids, polyunsaturated fatty acids, vitamins, pigments, phycobiliproteins, enzymes, etc. Biologically active substances from microalgae are capable of exhibiting antioxidant, antibacterial, antiviral, antitumor, regenerative, antihypertensive, neuroprotective and immunostimulating effects [3]. These compounds are in demand in pharmacology, medicine, cosmetology, the chemical industry, fish farming, the energy industry, agriculture in the production of feed and functional foods [4].

Microalgae are less studied than seaweeds, but their advantages are associated with rapid growth, high photosynthetic efficiency and the possibility of cultivation under production conditions. In addition, the biodiversity of microalgae will increase the number of different sources of biologically active substances, such as polysaccharides, lipids, proteins and pigments.

Currently, integrated technologies for the cultivation of microalgae aiming to isolate various biologically active substances from biomass to increase the profitability of algae production are being sought. The purpose of this review is to analyze recent works (less than 10 years) aimed at isolating biologically valuable substances from microalgae and assessing their biological activity. This review aims to assess the potential of microalgae as a raw-material basis for biologically valuable substances of various activity spectra.

## 2. Factors Affecting Biomass Production

The level of biomass accumulation and productivity of biologically active substances is an important indicator of the effectiveness of the microalgae strain. These parameters are influenced by many conditions, including the composition of the culture medium, temperature, pH, growth phase, method of harvesting and illumination [5].

The optimum growth temperature for the most commonly used microalgae, such as *Chlorella, Chlamydomonas, Botryococcus, Scenedesmus, Neochloris, Haematococcus* and *Nannochloropsis*, is in the range of 15–35 °C depending on the strain [6]. It has been shown that some microalgae strains have high stress resistance at high temperatures. For example, strains of *Asterarcys quadricellulare* and *Chlorella sorokiniana*, isolated from soil near a steel mill, not only grow at 43 °C, but are also resistant to high concentrations of CO_2_ and NO [7]. It is known that the use of thermotolerant microorganisms can significantly reduce the growth of incidental microflora. In addition, the use of such strains allows cultivation under natural conditions. The effect of technological parameters of the microalgae cultivation process (temperature, duration, stirring) on biomass production is presented in Table 1, Table 2 and Table 3.

The recommended cultivation temperature for maximum biomass yield of various types of microalgae is a temperature ranging from 27 °C to 30 °C. With increasing cultivation temperature to 35 °C, the biomass yield decreases sharply.

A significant accumulation of biomass was observed by the 14th day of cultivation of all the studied microalgae.

The growth of microalgae biomass under stationary conditions is slowed down compared to cultivation with stirring (50–100 rpm).

The hydrogen potential (pH) is of great importance in the cultivation of microalgae, as it determines the solubility of minerals and carbon dioxide in the medium, in addition to the direct effect on the microalgae themselves [8]. The pH of the culture medium can be influenced by such factors as composition and buffer capacity, the amount of dissolved carbon dioxide, temperature and metabolic activity of the cells. Different types of microalgae have different levels of tolerance to the pH of the culture medium, which may affect their growth rate. For some microalgae, the optimal pH ranges from 6 to 8. In a number of cases, a steady increase in the microalgae biomass is observed at extremely low pH values, as, for example, for *Chlorella protothecoides* var. *acidicola* isolated from the surface of the microbial mat and having a growth optimum at pH 2.5 and 30 °C [9]. The use of buffer solutions reduces pH fluctuations in cultures, however, for large-scale cultivation systems, the use of buffers increases the cost of production, making it impossible. One way to control pH changes is to aerate cultures by pumping atmospheric air (0.03% CO_2_) or CO_2_-enriched air through the medium, since carbon dioxide, when dissolved, reduces the pH of the medium [10]. The effect of pH on the biomass yield of various types of microalgae is presented in Table 4. The optimal pH indicator is in a range from 6 to 8, however, when the biomass of the microalgae *Chlorella vulgaris* is produced, a decrease in pH to 4 leads to the greatest yield.

Combining the given data, the recommended values of technological parameters for growing microalgae are presented in Table 5.

The salinity of the solution is another factor affecting the growth and development of microalgae. Some types of microalgae are very limited in terms of salinity, especially those found in freshwater. In general, microalgae can be divided according to salinity resistance into oligogaline when they can develop only in water with low salinity (maximum salinity 0.5–5.0 g/L), mesogaline—develop in media with moderately saltwater with a salinity of 5–18 g/L and polyhaline—develop in saltwater with a salinity of 18–30 g/L [11].

An ideal culture medium for microalgae should contain inorganic elements such as nitrogen (N) and phosphorus (P), which can vary depending on the cultivated species. After carbon, which is approximately 50% of the fraction of elements in the biomass of microalgae, nitrogen takes second place, with a concentration of 1% to 14% in the dry mass. It can be absorbed in inorganic forms of NO_3_, NO_2_, NO and NH_4_ and in some cases as N_2_ or in an organic form via urea or amino acids. The decrease in nitrogen concentration during cultivation leads to the predominant synthesis of lipids and polysaccharides. The phosphorus concentration in the dry biomass of microalgae can be from 0.05% to 3.3% [12]. In natural environments, as well as in wastewater, phosphorus is present in various forms, such as orthophosphate, polyphosphate, pyrophosphate and metaphosphate. In addition, there are various types of agricultural fertilizers that can be used to saturate the microalgae cultivation environment with phosphorus, such as phosphates and superphosphates derived from phosphorites. For adequate growth of microalgae, the medium must contain other nutrients—trace elements. The main trace elements are Mg, S, Ca, Na, Cl, Fe, Zn, Cu, Mo, Mn, B and Co, with an emphasis on Mg, S and Fe [13].

Nutrient limitation has a direct impact on the synthesis of biologically active substances, biomass growth and photosynthesis processes in microalgae [14]. High illumination and limitation of nutrients (nitrogen or phosphorus) lead to an increase in the size of the lipid fraction and stimulate the accumulation of triacylglycerols. In low light conditions, mainly polar lipids (phospholipids and glycolipids) accumulate, which are structurally and functionally associated with cell membranes. Cell growth is reduced under such conditions. However, there are exceptions, for example, diatoms, in which the lipid content in the long phases does not respond to nitrogen starvation [15].

Stress caused by nutrient deficiency also leads to the formation of free radicals in the cell and a change in antioxidant content. Primary carotenoids (chlorophylls, β-carotenoids, violaxanthin and voheriaxanthin) are synthesized under normal favorable microalgae growth conditions, especially in *Eustigmatophyceae strains*. However, secondary carotenoids are produced under stress caused by nitrogen deficiency, synthesized from primary carotenoids and accumulating outside the chloroplast after the cell growth phase [16]. Under intense exposure to light, microalgae are photo-damaged, and some become pale green and decrease in size, which indicates a decrease in the density of chlorophyll and affects cell development [17]. Furthermore, with excess light, violaxanthin is converted to zeaxanthin by removing epoxides using the anthraxanthin monoepoxycarotinoid. Zeaxanthin is epoxidized in low light conditions, and nitrogen deficiency contributes to the accumulation of astaxanthin pigment in some microalgae, such as *Haemotococcus pluvialis* [15].

During autotrophic growth, microalgae carry out oxygen photosynthesis and fix carbon dioxide. One part of fixed carbon is used to maintain cells and growth, while the other part is stored in several forms, depending on different types of microalgae. Microalgae require 1.8 to 2.0 kg of CO_2_ to produce 1 kg of biomass. Given this stoichiometric ratio, the amount of CO_2_ present in the air (0.03%) is not enough for high culture productivity. Thus, in order to increase the efficiency of photosynthesis, media with microalgae cultures should be supplemented with carbon, either in the form of salts, such as bicarbonate, or by introducing CO_2_-enriched air. A study by Dúran et al. [18] demonstrated that when the air was supplied (600 mL/min) to the photobioreactor, microalgae showed optimal growth with a CO_2_ content of up to 20% in the supplied air. This makes it possible to use CO_2_ from industrial combustion, generating on average 5.0% of CO_2_, in microalgae cultivation. This approach combines an inexpensive carbon source for microalgae and reduces CO_2_ emissions. The supply of CO_2_ to microalgae cultures can increase biomass productivity, however, a decrease in pH due to an increase in the availability of CO_2_ in the aqueous phase may impede the growth of certain types of microalgae [19].

The optimal conditions for the growth of microalgae depend on the light intensity, wavelength and photoperiod to which the cells are exposed during cultivation [20].

Light intensity is directly related to the photochemical phase of photosynthesis, when light is absorbed through chlorophyll molecules, the synthesis of adenosine triphosphate (ATP) and the photolysis of water. In general, photosynthesis can be defined as a process in which light energy synthesizes polysaccharides and oxygen from carbon dioxide and water [21]. Light intensity is one of the important aspects of growing microalgae and requires special attention. The amount of light received by cultured cells is directly related to carbon, which will be fixed, affecting the growth rate of cultures [22]. The light source can be either artificial or natural (sunlight), the latter being the most cost-effective due to its availability. Photosynthesis in microalgae increases with increasing light intensity until it reaches a maximum speed at the saturation point [23]. Above the saturation point, excess light leads to a phenomenon called photoinhibition, which is determined by the change and possible inactivation of photosystem II (PSII), affecting electron transport in the chain of reduction reactions from NADP^+^ to NADPH. Photoinhibition and photosynthesis can be classified as moderate or intense, which determines whether this inhibition is dynamic or chronic [24]. The light intensity does not affect its accessibility in the depths of the plant environment. In other words, even if the light intensity is high enough to cause inhibition of photosynthesis, the light may not reach the shaded cells, which affects the efficiency of biomass production. Therefore, to supplement natural light or even for cultures under artificial lighting, it is recommended that LEDs be placed inside the medium to improve photon delivery and distribution.

Photosynthetically active radiation, useful for microalgae, is in the range from 400 to 700 nm from light radiation, which corresponds to 50% of solar radiation and intensity from 800 to 1000 W/m^2^ [25]. This is confirmed by the results of Fu et al. [26], who demonstrated that *Dunaliella salina* shows the highest biomass and carotenoid productivity (β-carotene and lutein) when 75% of red light (wavelength about 700 nm) and 25% of blue light (wavelength about 400 nm) are used together, compared with the only red light.

There are light/dark (L/D) cycles in the microalgae growth tanks provided by the medium stirring system. These cycles affect the efficiency of photosynthesis conversion and biomass productivity in microalgae. It was also found that the efficiency of photosynthesis in *Chlamydomonas reinhardtii* improved when L/D cycles of less than 20 s were applied, with an increase in the growth rate of up to 40% [27].

Using the example of various types of marine and freshwater microalgae, it was shown that the duration of the photoperiod affects the intensity of photosynthesis, productivity, cell division rate and carbon dioxide consumption. Almost always, the listed indicators for various light and dark modes were higher than for continuous illumination. However, at present, there is no consensus on the mechanisms that explain the increase in productivity under the influence of the photoperiod [28].

There are several hypotheses in the literature that explain the phenomenon of an increase in the growth rate of lower phototrophs in the presence of a photoperiod. For example, under continuous illumination, some products of photosynthesis that inhibit it may accumulate in the cells, the outflow of which occurs in the dark period. Furthermore, in the presence of a photoperiod, the activity of ribulose-1,5-bisphosphate carboxylase may increase, which may be due to the regulation of carbon consumption mechanisms. It can be assumed that when limiting the growth of microalgae culture by CO_2_ concentration, the increase in productivity is due to an increase in carbon concentration during the dark period due to the dark respiration of the cells. Additionally, if there is a dark period in the daily cycle, a change in the biochemical composition of algae occurs, in particular, the respiratory consumption of reserve compounds, which, in turn, can lead to a decrease in respiration rate [29].

An effective stirring of the culture medium is important for obtaining high cell concentrations. Stirring maintains cells in suspension, eliminates thermal separation, distributes nutrients and increases gas exchange efficiency. Stirring can reduce the degree of self-darkening and the likelihood of photoinhibition by uniformly distributing light among all microalgae cells [30]. In addition, stirring is also responsible for the possibility of CO_2_ capture from the atmosphere and facilitates the transfer of biosynthesized O_2_ from the liquid phase to the gaseous one, which stimulates photosynthesis in microalgae culture [31]. The connection between stirring and illumination becomes more obvious when the culture has a high concentration of cells, since under this condition the light is blocked by cells of the surface region and the light intensity decreases sharply with the depth of the culture [32]. In a study by Sánchez et al. [33], it can be noted that in cultivation systems with stirring blades, the daily growth of the microalgae culture *Isochrysis galbana* was two times higher compared to a system without stirring (8.8 × 10^5^ and 4.0 × 10^5^ cells/mL).

The growth rate of photosynthetic microorganisms increases with increasing turbulence caused by stirring, but above the optimum level of turbulence, this growth decreases sharply due to damage to the cells. Many photosynthetic microorganisms have a fragile cell wall; some are mobile or threadlike and may be subject to physical exertion. Thus, it is desirable that the stirring is carried out with the lowest possible hydrodynamic stress.

## 3. Methods of Microalgae Cultivation

The conditions for the cultivation of microalgae can be divided into three main methods: photoautotrophic, heterotrophic and mixotrophic. Photoautotrophic is the most commonly used cultivation method. Microalgae use light (usually solar) as an energy source and inorganic carbon (e.g., carbon dioxide) as a carbon source to generate chemical energy. With heterotrophic cultivation, microalgae can grow not only under photoautotrophic conditions, but also use organic carbon in the absence of light. Owing to the ability to grow in the dark, microalgae growing during heterotrophic cultivation are much less demanding on the ratio of surface area to volume than autotrophic cultivation [34].

Mixotrophic cultivation is a generalized two-stage mode in which microalgae have a high initial content of organic carbon, but are induced for assotrophic assimilation of CO_2_ due to depletion of organic substances and oxygen production through photosynthesis [35,36]. During mixotrophic cultivation, microalgae grow under optimal autotrophic and heterotrophic conditions, combining the advantages of both cultivation modes [37]. Under such conditions, acetyl-CoA is produced and maintained both by fixing CO_2_ and extracellular organic carbon, which indicates a decrease in photoinhibition. Mixotrophic cultures are often observed in ecological water bodies, where they are supported by both chemical, physical and organic activity of biota. Venkata Mohan et al. [38] presents in detail metabolic schemes for each cultivation mode.

The design and operation of an algae cultivation system play a decisive role in both the productivity of photosynthesis and economic efficiency. The design of the cultivation system should have a short path of light, the optimal volume of liquid for appropriate stirring and scattering of light. Working with large volumes of fluid requires high hydrostatic pressure and higher energy consumption for proper stirring [39]. Various large-scale cultivation systems have been developed for the large-scale production of microalgae. They usually work under photoautotrophic conditions and can be divided into two main groups: open or closed cultivation systems. Ninety percent of microalgae biomass production worldwide is through open cultivation systems. Open systems usually use open shallow ponds made of aligned raceways (2–10 m wide and 15–30 cm in depth) in the form of simple loops or meandering systems where turbulence is provided by rotating impellers [40]. These reactors are easily expandable, and investment and operating costs are relatively small. The main disadvantages of open cultivation systems are the complexity of process control, as well as the inability to organize a continuous cultivation process. High susceptibility to contamination by other microalgae and microorganisms, dependence on weather conditions (light, temperature) and daily temperature fluctuations make the open pond system unsuitable for large-scale culture [35,41,42].

Closed photobioreactors were designed to overcome the aforementioned disadvantages of open pond systems, resulting in a series of reactor designs that increased surface-to-volume ratio and system reliability [32,43]. Closed systems allow continuous operation and offer higher biomass productivity and quality, as well as higher photosynthesis efficiency. Liquid evaporation is minimized through the use of a closed system. However, such systems are difficult to scale due to their complex organization and reduction of light penetration with increasing cell concentration and the removal of oxygen generated during photorespiration is a serious problem. Closed photobioreactors are certainly more expensive than open systems [44].

There are bubble-airlift, bubble-column, tubular and panel photobioreactors. Mostly in pilot tubular photobioreactors, microalgae grow at a depth of illumination of 3–6 cm, where light penetration is less than 50%. Panel photobioreactors are the simplest solution for laboratory-scale microalgae cultivation. Of all these types of photobioreactors, they have a maximum illuminated surface and allow achieving high cell densities. However, during their operation, it is necessary to maintain a balance of illumination to prevent photoinhibition. The main achievements in the field of closed photobioreactors have been considered by Wang et al [45].

Currently, several basic technologies for the production of microalgae biomass are being developed in Russia and abroad, which include cultivation: (a) in open waters; (b) in greenhouse ponds; (c) in closed photobioreactors. Genomic and biochemical technologies are used for microalgae cultivation. In the framework of this work, the effect of all three types of the described methods for the cultivation of microalgae was studied [46]. The characteristics of the possible methods of microalgae cultivation under various conditions are presented in Table 6.

The production of microalgae remains an expensive process, and therefore efforts are being made to develop more efficient photobioreactors, as well as to optimize their control and modeling in order to maximize biomass yield and reduce production costs [46]. Each of the considered methods has both advantages and disadvantages, however, the heterotrophic method is the most preferred method for the cell biomass accumulation.

## 4. Microalgae Proteins Extraction Methods

The total protein content in biomass depends on the type of microalgae and varies from 30% to 70% of dry weight [47]. It has been reported that some microalgae contain soluble proteins in their cytoplasm. In addition, chloroplast microalgae contain soluble protein, central pyrenoid and phytobiliproteins, although some microalgae, such as *Arthrospira plantesis*, *Chlamydomonas reinhardtii* and *Neochloris cohaerens* instead have thylakoid sacs surrounding the peripheral part of the cytoplasm associated with phycobilisomes [48].

The amino acid composition of microalgae proteins is quite diverse and is not inferior in biological value to animal proteins [49]. The amino acid profile of *Arthrospira platensis* includes leucine, valine, isoleucine, phenylalanine, methionine, cysteine and tyrosine. The *Porphyridium cruentum*, *Haematococcus pluvialis* and *Nannochloropsis gaditana* microalgae proteins contain aspartic acid, threonine, serine, glutamic acid, glycine, alanine, cysteine and valine. In diatom microalgae, the main amino acids are serine, alanine, arginine, leucine, glycine, aspartate and threonine. In *Chlorella vulgaris*, *Botryococcus braunii* and *Scenedesmus obliquus* the essential amino acids cysteine and arginine account for about 44.7% of the total amino acid profile [50,51].

Vernèsa et al. [52] suggested a new method for the extraction of proteins from *Arthrospira platensis*, based on the combined effect of three parameters: pressure, temperature and ultrasound. Using the developed method, the authors were able to increase the protein yield by 229% compared with the usual method of ultrasonic treatment. It is assumed that the combined effect of pressure, temperature and ultrasound better destroys cells and intensifies the mass transfer process compared to using only ultrasound. Acoustic cavitation acted on *Arthrospira platensis* filaments through various mechanisms, such as fragmentation, sonoporation and destruction. These phenomena contributed to more efficient extraction of proteins from *Arthrospira platensis*.

Xie et al. [53] studied the effect of the concentration of nitrates in the nutrient medium on the accumulation of protein in microalgae *Chlorella vulgaris*. The authors were able to increase the protein content to 44.3% under optimized cultivation conditions.

Many methods of concentrating and isolating proteins from microalgae are difficult to scale. The three-phase separation method has attracted the interest of many researchers due to its quick, simple and scalable use for the concentration, isolation and deactivation of proteins from crude samples. Waghmare et al. [54] studied the effect of various parameters on the three-phase separation method to optimize the process of protein isolation from *Chlorella pyrenoidosa*. Also, Chia et al. [55] presented the extraction of protein from the microalgae *Chlorella vulgaris* by the method of three-phase separation with ultrasonic treatment. As a result of using additional ultrasonic processing, the authors were able to obtain an increased protein yield in a shorter period of time. Three-phase ultrasound separation is believed to be a more efficient method for extracting biomolecules from microalgae.

Safi et al. [47] analyzed the effectiveness of aqueous extraction of proteins from five microalgae (*Haematococcus pluvialis, Nannochloropsis oculata, Chlorella vulgaris, Porphyridium cruentum, Arthrospira platensis*) using various methods of cell destruction. It was found that the highest yield of protein occurs after the destruction of cells under high pressure using water extraction, followed by chemical, ultrasonic and mechanical treatments. Lupatini et al. [56] presented a method for extracting proteins and polysaccharides from *Spirulina platensis* biomass using the ultrasonic treatment and mechanical stirring under alkaline conditions. Under optimized extraction conditions, when treated with ultrasound for 33–40 min and stirring for 40–55 min, the protein yield was 75.76% and polysaccharides yield was 41.52%. Safi et al. [57] presented mild bioprocessing of the microalga *Nannochloropsis gaditana* to obtain a water-soluble protein fraction free of chlorophyll. To destroy the cells, homogenization under pressure or enzymatic hydrolysis was used, followed by ultrafiltration/diafiltration. Kose et al. [58] obtained protein hydrolysates from the microalgae *Chlorella vulgaris, Chlorella cyanobacterium* and *Spirulina platensis* and evaluated their digestibility in in vitro experiments. The studied hydrolysates showed rather high digestibility values and low cytotoxicity.

Nee et al. [59] evaluated the effect of various types of solvents (methanol, ethanol, 1-propanol and water) on the yield of proteins from the cell wall of microalgae. It was found that water is the most effective extractant of microalgae proteins compared to other solvents.

Data analysis (Table 7) reveals the prospects for the use of combined approaches (for example, simultaneous flocculation and filtration) for the isolation of proteins from the biomass of cells of all types of microalgae.

## 5. Microalgae Lipids

Microalgae can produce various types of lipids: triacylglycerols, phospholipids, glycolipids or phytosterols, which contain fatty acids ranging from C12 to C24, often with mono- and poly-unsaturated fatty acids C16 and C18. The lipid content in microalgae varies from 20% to 50% of dry weight. These lipids can be used for energy storage, as energy substrates, as structural components of the cell membrane and for metabolic processes (signal transduction, transcriptional and translational control, intercellular interactions, secretion and transfer of vesicles) [60]. The number of lipids and the presence or position of double bonds in the carbon chain may vary depending on the type of microalgae and cultivation conditions. Optimal conditions can facilitate the conversion of fatty acids to glycerol-based membrane lipids, while adverse conditions can increase the synthesis of neutral lipids, such as triacylglycerols. Typically, many microalgae contain polyunsaturated fatty acids (PUFAs) such as arachidonic acid, eicosapentaenoic acid and docosahexaenoic acid. The main saturated fatty acid is palmitic [5]. The importance of lipids derived from algae lies in their commercial value as an alternative source for obtaining functional food products from their PUFAs, such as eicosapentaenoic acid (EPA) and docosahexaenoic acid (DHA), as well as their precursor α-linolenic acid [60]. Most microalgae with high omega-3 content are marine species, for example, *Schizochytrium* sp. and *Nannochloropsis* sp. However, freshwater species, for example, *Desmodesmus* sp., have also been investigated as a source of omega-3 long-chain PUFA, EPA and DHA acids [61,62]. It was found that freshwater microalgae species produce biomass with a lower amount of PUFA.

Since the human body is not able to produce some essential fatty acids, they must be obtained from food or using various food additives, often obtained from fish and fish oil. However, due to the increased interest in the modern world for vegetarian and vegan diets, microalgae can become an alternative source of these nutritional supplements. In addition, many types of fatty fish contain traces of heavy metals that can adversely affect health and cause neurotoxic effects. Depending on the cultivation conditions and the composition of the nutrient medium, the profile of fatty acids may vary for the same types of microalgae. For example, Scharff et al. [63] evaluated the effect of the photoperiod on the biochemical profile of microalgae *Chlorella vulgaris* and *Scendesmus obliquus* and found that longer photoperiods (24:0, 22:2, 20:4) can reduce the synthesis of α-linolenic acid and cause the synthesis of linoleic acid, which is more distinctly observed for *C. vulgaris* than *S. obliquus*. On the other hand, Chandra et al. [64] established that the content of both linoleic and α-linolenic acids increases together with an increase in light intensity, which is a continuous lighting condition for an optimal photoperiod. Choi et al. [65] studied the dependence of biomass productivity and total fatty acid content in microalgae on various lighting conditions (continuous light, light-dark cycle, continuous darkness and continuous darkness with additional flashing light). The analysis found that the use of flashing light significantly increased the growth rate of *Chlorella vulgaris* and the concentration of fatty acids in the biomass. Carpio et al. [66] estimated the lipid content and profile in the biomass of the microalgae *Chlorella vulgaris Beij* at various concentrations of Fe and CO_2_. It was found that the most intensive growth of biomass (460.0 ± 10 mg/L of dry biomass) was observed at a concentration of Fe (4.8 × 10^−5^ mol Fe/L) with 2% CO_2_. While the total lipid content (27.0 ± 0.8%) was maximum at a concentration of Fe (2.4 × 10^−5^ mol Fe/L) and 2% CO_2_. Thus, we can conclude that the concentration of iron and other elements also affects the total lipid content, which can significantly affect the profile of fatty acids [67]. Abd El Baky et al. [68] studied production, lipid accumulation, and the profile of fatty acids in the microalgae *Dunaliella salina*. By varying the CO_2_ aeration from 0.01% to 12.0%, a 20-fold increase in the lipid content in *Dunaliella salina* was obtained. Ramirez-Lopez et al. [69] suggested a new culture medium stimulating the growth of biomass and lipid accumulation for the microalga *Chlorella vulgaris*. In the process of optimizing the component composition of the medium, the contents of sodium nitrate, ammonium bicarbonate, heptahydrate of magnesium sulfate, potassium dihydrogen phosphate, dipotassium phosphate and diammonium phosphate were varied. Using a new culture medium, it was possible to increase biomass productivity by 40% and an 85% lipid concentration. The concentration of some components of the nutrient media was reduced to 50%. Josephine et al. [70] studied the influence of growth factors and the optimization of the collection time of *Chlorella vulgaris* to increase lipid production. An exogenous supplement called the chlorella growth factor (CGF) significantly increased biomass and lipid levels, which determined its potential as a biomass growth promoter in large-scale production. It was found that nitrogen starvation favored the synthesis of more unsaturated fatty acids than saturated ones.

Cultivation of *Chlorella vulgaris* in the presence of sodium selenite and chromium (III) chloride yielded a biologically active lipid complex, including selenium and chromium [71]. The authors evaluated the effect of the obtained complex on the energy metabolism of rats subjected to experimentally induced diabetes mellitus. It was found that in rats, the selenium-chrome-lipid complex from the *Chlorella* improved energy metabolism. The authors concluded that this complex in comparison with inorganic forms of chromium and selenium has potential for regulating energy metabolism in people with diabetes. It was found that the energy metabolism of rats increased when using a biologically active lipid isolated from the microalgae biomass.

Anthony et al. [72] presented a column chromatography technique for sequential isolation of several biologically active substances from the *Chlorella vulgaris* biomass (a nucleotide-peptide complex enriched with vitamins, minerals and polysaccharides, lipids and carotenoids). The proposed method [70] allowed to obtain an increased yield of lipids (18%) and lutein (9%) without foreign impurities of chlorophyll. The highest lipid content was noted in microcultures of microalgae *Haematococcus pluvialis, Scenedesmus obliquus* and *Chlorella vulgaris*. The amount of lipids in these microalgae cultures is 19.61 ± 0.58%, 17.13 ± 0.51% and 16.24 ± 0.48%, respectively. The fat content in the *Neochloris cohaerens* cell culture was 6.61 ± 0.19%, which is 1.35 times higher than the lipid content in the dry biomass of *Chlamydomonas reinhardtii* microalgae (4.90 ± 0.14%), but less than in the microbial cell culture of *Botryococcus braunii* and *Nannochloropsis gaditana* (7.23 ± 0.21% and 7.84 ± 0.13%, respectively). Further study of the microbial lipid profile of microorganisms *Chlorella vulgaris, Botryococcus braunii, Neochloris cohaerens, Chlamydomonas reinhardtii* and *Nannochloropsis gaditana* was carried out using gas chromatography. A reagent for transesterification of triglycerides of fatty acids was preliminarily prepared. The studied samples of microalgae (*Chlorella vulgaris, Botryococcus braunii, Neochloris cohaerens, Chlamydomonas reinhardtii* and *Nannochloropsis gaditana*) were characterized by a diverse fatty acid composition (Table 8). It was found that the fatty acid composition of the studied microcultures is represented by high-molecular-weight polyunsaturated fatty acids.

## 6. Microalgae Polysaccharides

It has been reported that polysaccharides of the microalgae *Chlorella sorokiniana* can induce the secretion of interleukin 12 (IL12), which activates natural killer cells (NKs) and leads to the differentiation of T helper cells into Th1 cells. This effect is important in antiviral and antitumor therapies [73].

Song et al. [74] selected parameters for the extraction and purification of the polysaccharide from the Arctic strain of *Chlorella* sp. to evaluate its antioxidant activity. Under optimized conditions, the polysaccharide yield was 9.62 ± 0.11% of dry weight. After its purification, three fractions were obtained: PI, P-II and P-III. The highest antioxidant activity was shown by the P-IIa fraction. Structural analysis showed that P-IIa is a spiran group heteropolysaccharide consisting mainly of rhamnose, arabinose, glucose and galactose.

Liu et al. [75] presented a method for producing a new type of polysaccharide from the microalga *Arthrospira platensis*. Microalgae were cultured under conditions of nitrogen deficiency in open industrial reservoirs. The maximum productivity of biomass and polysaccharides was 27.5 g/m^2^·day and 26.2 g/m^2^·day, respectively. The polysaccharide was extracted with hot water by homogenization under pressure, and purification was carried out by flocculation.

El-Ahmady El-Naggar et al. [76] extracted and identified water-soluble polysaccharides from the microalga *Chlorella vulgaris* in order to use them as plant growth stimulants.

Liu et al. [77] isolated a water-soluble polysaccharide from *Haematococcus pluvialis* using a DEAE-52 anion exchange column and Sephacryl S400 chromatography. The resulting polysaccharide showed immunomodulatory activity.

Gaignard et al. [78] investigated 166 species of marine microalgae and cyanobacteria in order to identify strains producing original exopolysaccharides. Forty-five strains with the desired characteristics were isolated. Eight new genera of microalgae producing exopolysaccharides, including polymers with a very original composition, were discovered.

In the course of the studies, it was found that the microalgae cultures of *Botryococcus braunii* and *Haematococcus pluvialis*, in comparison with other microalgae cells, are characterized by a high content of carbohydrates: 27.36 ± 0.76% and 21.95 ± 0.74%, respectively. The mass fraction of carbohydrates in the biomass of microalgae *Scenedesmus obliquus* reaches a value of 13.69 ± 0.34, and the carbohydrate content in the biomass of microalgae *Nannochloropsis gaditana* is 15.34 ± 0.51. Microalgae *Chlamydomonas reinhardtii, Neochloris cohaerens* and *Chlorella vulgaris* synthesize carbohydrates in the amount of 12.48 ± 0.34%, 12.58 ± 0.34% and 12.23 ± 0.33%, respectively.

## 7. Microalgae Pigments and Vitamins

Microalgae contain various types of pigments: carotenoids (orange color), xanthophylls (yellowish tint), phycobilins (red or blue color) and chlorophylls (green color). The content of carotenoids and chlorophyll in microalgae is usually higher than in some plants [79].

Carotenoids can be stored in oil droplets, in the stroma of the chloroplast or the cytosol, depending on the type of microalgae. Carotenoids in microalgae are usually present in low concentrations (0.5% μg^−1^ dry weight), although in some *Chlorophyta* they can reach up to 10% μg^−1^ dry weight when cultivated under adverse conditions, as is the case for *Dunaliella salina* [80]. Carotenoids play an important role in oxygen photosynthesis, as a direct quencher of reactive oxygen species, as well as in the thermal dissipation of excess energy in the photosynthetic apparatus. Microalgae carotenoids can also be used as antioxidant molecules that can quench free radicals, thereby protecting cells and tissues from oxidative damage, including preventing oxidative spoilage of food products. The main carotenoids of microalgae are β-carotene, lycopene, astaxanthin, zeaxanthin, violaxanthin and lutein. Among them, β-carotene, lutein and astaxanthin are the most studied [81].

Phycobilins (phycocyanin and phycoerythrin) are found in the stroma of the chloroplasts *Cyanobacteria, Rhodophyta, Glucophyta* and some cryptomonads. They are highly soluble in water and are widely used in the food industry as dyes and in molecular biology as fluorescent markers [79].

Chlorophylls are fat-soluble green pigments that are actively involved in the process of photosynthesis [82]. The chlorophyll content in microalgae varies from 0.5% to 1.0% of dry weight, mainly chlorophyll a, however, some microalgae, for example, *Dinophyta* also contain chlorophyll b and c [81].

Microalgae also represent a valuable source of vitamins: A, B1, B2, B6, B12, C, E, biotin, folic acid, pantothenic acid, etc. For example, *Isochrysis galbana* is an important source of vitamins A and E, folic acid, nicotinic acid, pantothenic acid, biotin, thiamine, riboflavin, pyridoxine, cobalamin, chlorophyll (a and c), fucoxanthin and diadinoxanthin, while *Euglena gracilis* antioxidant can produce vitamins such as β-carotene and vitamins C and E [80].

Vitamin B12 refers to water-soluble vitamins and is synthesized in animal products, but absent in plant ones. Vitamin B12 deficiency is common in people following a vegan or vegetarian diet. Some types of microalgae may contain or synthesize vitamin B12, for example, *Chlorella* sp. and *Pleurochrysis carterae*. However, it is not always in bioavailable form and further studies are needed to identify potential sources of vitamin B12 among microalgae [83].

Vitamin E is synthesized in many microalgae, for example, *Dunaliella tertiolecta, Tetraselmis suecica, Nannochloropsis oculata, Chaetoceros calcitrans* and *Porphyridium cruentum*. A number of studies indicate that the content of vitamin E in microalgae can be much higher than in plants. In this regard, microalgae are a valuable source for vitamin E production [84].

Smerilli et al. [85] studied the dependence of the vitamin C content in the microalga *Skeletonema marinoi* on the spectral composition and light intensity. It has been found that with an increase in the intensity of ultraviolet light, the content of vitamin C in microalgae increases.

Papadaki et al. [86] extracted various biologically active substances from microalgae *Haematococcus pluvialis* and *Phaeodactylum tricornutum* using ultrasound (to restore β-carotene, astaxanthin, fucoxanthin, eicosapentaenoic and arachidonic acid from selected microalgae, coconut oil was used as solvent). The yield of the targeted biologically active substances (vitamins E, B, C and pigments) reached about 80%.

Grudzinski et al. [87] studied the mechanism of the adaptive response of microalgae (*Chlorella protothecoides* and *Chlorella vulgaris*) to the effect of intense illumination (400 μmol photons m^−2^ s^−1^). It has been established that the color change of microalgae from green to yellow under the influence of intense illumination is associated with the accumulation of xanthophilic pigments, mainly zeaxanthin. The results obtained indicate that the carotenoids synthesized in response to intense illumination are not energetically associated with chlorophylls and are not photosynthetically active. These pigments can potentially be used as antioxidants, they stabilize the membrane and protect cells from intense light.

Soares et al. [88] investigated various methods for the extraction of lutein and β-carotene from the microalga *Desmodesmus* sp. When choosing the best extraction method, various types of solvents were analyzed and also their proportional ratio under ultrasonic extraction conditions, extraction efficiency, the effect of pretreatment on the yield of the target product, the stability of the extracts and the assessment of the qualitative and quantitative profile of pigments.

Pataro et al. [89] evaluated the effect of pretreatment by pulsed electric fields on the efficiency of the release of carotenes and chlorophyll a from the microalgae *Nannochloropsis oceanica* using supercritical CO_2_ extraction. The joint use of these processes contributed to a significant increase in the yield of the studied pigments.

Singh et al. [90] studied the effect of stressful conditions on the carotene yield of *Dunaliella salina* microalgae and evaluated the antioxidant and cytotoxic activity of the obtained carotene-enriched extracts. Stress conditions were created by varying the concentrations of salt, nitrogen and the temperature of cultivation. It was shown that *D. salina* grown under various stress conditions (varying NaCl concentration and temperature) increases carotene production. An increase in antioxidant and cytotoxic activities is caused by the accumulation of carotenes.

Sathasivam et al. [91] selected the optimal concentrations of NaCl and KNO_3_ to intensify the synthesis of β-carotene in the microalga *Dunaliella salina* KU11. The highest yields of β-carotene (115.5 ± 0.4 μg/mL) were obtained by creating stress conditions for the concentration of salts with a concentration of 2.5 M NaCl and 0.5 g/L KNO_3_.

The pigment complex of microcultures includes carotenoids and chlorophylls a and b [88]. Depending on the type of microculture and the composition of the nutrient medium, the quantitative content of the pigment complex may vary. The main part of the pigments of the studied microalgae samples (*Chlorella vulgaris*, *Chlamydomonas reinhardtii*, *Botryococcus braunii*, *Scenedesmus obliquus*, *Neochloris cohaerens*, *Haematococcus pluvialis* and *Nannochloropsis gaditana*) are chlorophylls. Analysis of the data (Table 9) allows conclusion that the content of chlorophyll a is predominant in the pigment complexes of microalgae; however, for the microalga *Scenedesmus obliquus*, the content of chlorophyll b is 1.85-times higher than that of chlorophyll a.

It was found [79,80,81] that in the microalgae *Botryococcus braunii*, *Neochloris cohaerens*, *Chlamydomonas reinhardtii*, *Nannochloropsis gaditana* and *Chlorella vulgaris*, the content of chlorophyll a exceeds the quantitative content of chlorophyll b. Therefore the microalgae *Botryococcus braunii* contains 6.58 ± 0.19 mg/g and 2.17 ± 0.07 mg/g of chlorophyll a and b, respectively. Microalgae cells of *Neochloris cohaerens* contain 3.9-times less chlorophyll b (6.13 ± 0.18 mg/g and 1.57 ± 0.05 mg/g). Cultures of the genus *Chlamydomonas reinhardtii* are characterized by a content of 2.53 ± 0.07 mg/g and 1.72 ± 0.04 mg/g of chlorophyll a and b, respectively. The microculture of Nannochloropsis gaditana is slightly inferior to the microculture of the microalga *Chlorella vulgaris* in the content of chlorophyll a, but not inferior in the content of chlorophyll b.

## 8. The Biological Activity of Various Substances from Microalgae

This section provides a brief analysis of the works aimed at assessing the biological effects of various substances from microalgae.

### 8.1. Antitumor Activity

Oncological diseases are one of the main causes of death in the modern world [92,93]. In recent years, studies on the search for new substances with antitumor properties have become increasingly relevant. Alginates, fucoidans, zosterol, unique sulfated polysaccharides, enzymes and peptides of microalgae possess antitumor activity [94].

Lauritano et al. [95] analyzed the antitumor properties of extracts from 32 species of microalgae against human melanoma cells A2058. Different types of algae were grown under three different cultivation conditions, the studied extracts were tested for possible antioxidant, anti-inflammatory, antitumor, antidiabetic, antibacterial and antibiofilm activities. One clone of the *S. marinoi* species (FE60) exhibited antitumor activity on human melanoma cells A2058, but only when cultured under nitrogen starvation. Another clone, FE6, was not active against cancer cells. Both clones (FE60 and FE6) exhibited antibacterial properties, inhibiting the survival of *S. aureus*. However, FE60 had antibacterial properties only when cultured under nitrogen starvation conditions, and FE6 only under phosphate starvation conditions.

Somasekharan et al. [96] evaluated the antitumor activity of an aqueous extract of microalgae from Canadian waters against various human cancer cell lines, including lung, prostate, stomach, breast, pancreas cancer and osteosarcoma. The authors analyzed the ability of microalgae extract to inhibit the formation of colonies in cancer cells. In vitro microalgae extract showed pronounced anticolony-forming activity.

Chen et al. [97] extracted sulfated polysaccharides from filamentous microalgae *Tribonema* sp. and tested their activity on liver cancer cells, HepG2. The MTT test showed that the sulfated polysaccharide from *Tribonema* sp. possessed antitumor activity, causing induced cell apoptosis.

Samarakoon et al. [98] studied the antitumor activity of the alcoholic fatty acid ester synthesized by the microalgae *Phaeodactylum tricornutum* Bohlin. Antitumor activity was evaluated for three different cancer cell lines (human leukemia (HL-60), human lung cancer (A549) and mouse melanoma (B16F10)). The strongest suppression of cancer cell growth was observed in HL-60 (IC_50_ = 65.15 μM) compared with other cancer cells in vitro.

Jabeen et al. [99] estimated the effect of enzymatic treatment on the antitumor activity of microalgae extracts. Before extraction, the microalgae cells were treated with cellulase and lysozyme. The antitumor activity of the extracts was evaluated against four cancer cell lines (A549, MCF-7, MDA MB-435, LNCap).

### 8.2. Antimicrobial Activity

Recently, more and more research has been aimed at finding alternative antimicrobial agents, in connection with the progressive problem of microbial resistance, arising from the widespread use of antibiotics in the modern world. Microalgae are able to synthesize a number of metabolites with antimicrobial, antiviral and antifungal properties. In addition, some microalgae can grow under quite extreme conditions, which leads to the synthesis of unique substances for adaptation to changing environmental conditions. Thus, the content of biologically active substances directly depends on the environmental conditions of cultivation. It is known that many algae can synthesize a chemical defense system in order to survive in a competitive environment. Lutein and ferulic acid, polyunsaturated fatty and organic acids, active metabolites and other unique biologically active substances isolated from microalgae exhibit antimicrobial activity [100].

Krishnakumar et al. [101] estimated the antimicrobial activity of biologically active metabolites produced by the microalgae *Dunaliella salina*. It was found that the highest antimicrobial activity was shown by biologically active substances extracted with a mixture of chloroform and methanol.

Kilic et al. [102] studied the influence of environmental components and cultivation conditions on the synthesis of antimicrobial substances by microalgae of the genus *Dunaliella*. The most productive *Dunaliella* species (*Dunaliella* sp. 1–4) with high biomass yield and the most resistant to various pollutants were selected. It was established that the highest antimicrobial activity was exerted by biologically active substances produced by microalgae *Dunaliella* sp. 2. The optimal conditions for the cultivation of biomass for the synthesis of biologically active substances with the highest antimicrobial activity (1.0 g/L nitrogen, 20% (weight/volume) NaCl, light intensity 4800 lux, cultivation time 14 days) were selected. Various types of solvents were analyzed (ethanol, methanol, hexane, chloroform, Tris-HCl and water) for the extraction of biologically active substances. The highest antimicrobial activity among the studied solvents was exerted by biologically active substances extracted with chloroform. When analyzing the obtained biologically active substances, it was suggested that lutein and ferulic acid were the main compounds responsible for higher antimicrobial activity under stressful conditions. The authors view *Dunaliella* sp. 2 as a safe biomaterial and recommend its use in the field of pharmacology in accordance with its biologically active properties.

### 8.3. Antioxidant Activity

The antioxidant is a biological molecule that protects the body or vital compounds from oxidative processes under the influence of radicals [103]. Many natural antioxidants are often included in various cosmetics as an active ingredient and to protect their components from oxidative processes. Antioxidants are also actively used in the production of functional foods [104,105]. Microalgae, as photosynthetic organisms, are often exposed to reactive oxygen species, as a result of which they can accumulate various antioxidant complexes and have developed a mechanism for protecting cells from the action of free radicals. The wide variety of species, the possibility of modulation of growth and simplicity of cultivating microalgae mean they can be considered one of the promising natural resources for the production of antioxidant compounds. Caratinoids, dimethyl sulfoxide, unique phenolic and nitrogen compounds isolated from microalgae possess antioxidant activity of microalgae [106].

Widowati et al. [107] investigated the antioxidant activity of methanol extracts from three different microalgae: *Dunaliella salina, Tetraselmis chuii* and *Isochrysis galbana clone* Tahiti. All three microalgae showed high antioxidant activity. *Dunaliella salina* and *Tetraselmis chuii* showed the best result. The inhibition rate in *D. salina* was 62.19% at a concentration of 500 ppm, and *T. chuii* showed the best result (71.36%) at a concentration of 1000 ppm.

Phenolic compounds play an important role in the classification of antioxidants in plants. However, the role of phenolic compounds as antioxidants in microalgae remains unknown. Gürlek et al. evaluated the antioxidant activity of a number of phenolic compounds obtained from *Galderia sulphuraria, Neochloris texensis, Stichococcus bacillaris, Ettlia carotinosa, Chlorella minutissima, Schizochytrium limacinum, Crypthecodinium cohnii* and *Chlorella vulgaris* microalgae extracts [3]. The maximum antioxidant activity and the content of phenolic compounds were detected in the *Galderia sulphuraria* extract. A high correlation coefficient between antioxidant activity and the content of phenolic compounds was established—in this regard, we can assume that the antioxidant activity of microalgae can be due to the presence of phenolic compounds in their composition.

Blagojevic et al. [108] studied the effect of nitrogen content on the antioxidant activity and profile of phenolic compounds in ethanol extracts of ten strains of cyanobacteria. As promising producers of antioxidants and phenolic compounds, the authors recommended cyanobacteria: *Nostoc, Anabaena* and *Arthrospira*.

Agregán et al. [109] evaluated the antioxidant potential of extracts obtained using ultrasound from microorganisms *Chlorella* and *Spirulina*. It was found that extracts of the studied microalgae as sources of phenolic antioxidants are more suitable for use as a component of human food. The relatively low antioxidant potential (in terms of polyphenols) makes microalgae extracts unsuitable for industrial use, unlike macroalgae.

Dantas et al. [110] evaluated the antioxidant and antibacterial activity of various extracts from the microalgae *Scenedesmus subspicatus*. The studied extracts were able to inhibit the growth of *Bacillus subtilis*. However, only dimethyl sulfoxide (as an extractant) inhibited the growth of *Klebsiella pneumoniae* and *Escherichia coli*. Obtaining an aqueous extract (the presence of antioxidant effectiveness has been proven) is an economical method and avoids the use of toxic substances.

Sansone et al. [111] analyzed the biological activity of the aqueous-alcoholic extract of the microalgae *Tetraselmis suecica* containing high concentrations of carotenoids. The studied extract had a high antioxidant and reparative activity.

Bioactive peptides obtained after protein hydrolysis can have various beneficial effects. Hu et al. [112] found that enzymatic hydrolysates of microalgae exhibit antioxidant properties. Chen et al. [113] evaluated the hepatoprotective effect of the antioxidant peptide obtained by enzymatic hydrolysis from the microalgae *Isochrysis Zhanjiangensis* on alcohol damage to HepG2 cells.

### 8.4. Anti-Inflammatory Activity

An inflammatory reaction is a pathological process that occurs in response to damage, irritation or injury. Regardless of what was the first stimulus, the classic inflammatory response includes pain, fever, redness and swelling. Microalgae substances with a unique structure and properties, such as phycocyanin, polysaccharides, monosaccharides, enzymes, polyunsaturated fatty acids, peptides, and polyphenols have anti-inflammatory properties [114].

*Spirulina (Arthrospira platensis)* is a highly nutritious blue-green microalgae widely used worldwide as a nutraceutical food supplement. In addition to its nutritional value, it also exhibits therapeutic properties, including anti-inflammatory activity. *Spirulina* contains a unique component, phycocyanin, which inhibits the formation of pro-inflammatory cytokines such as TNFα, reduces the production of prostaglandin E(2) and inhibits the expression of cyclooxygeanase-2 (COX-2) [115]. β-carotene, another compound present in *Spirulina*, the accumulation of which suppresses the transcription of IL-1β, IL-6 and IL-12, inflammatory cytokines, in the macrophage cell line stimulated by lipopolysaccharide or IFNγ [116].

*Spirulina* biomass also promotes the growth of probiotic bacteria such as *Lactobacillus casei, Streptococcus thermophilus, Bifidobacteria* and *Lactobacillus acidophilus*. Such prebiotic properties of microalgae are caused not only by the presence of polysaccharides in their structure, but also by monosaccharides, enzymes, PUFAs, peptides and polyphenols [117].

Extracts of *Phaeodactylum tricornutum* and *Chlorella stigmatophora* also exhibit anti-inflammatory properties along with some analgesics and antioxidants [118].

### 8.5. Antiplatelet Activity

Cardiovascular diseases remain the main global cause of death, which indicates the need to identify all possible factors that reduce primary and secondary risk.

Villar et al. [119] found that the crude aqueous extract of the microalga *Dunaliella tertiolecta* has a strong inhibitory effect on human platelet aggregation caused by thrombin, arachidonic acid and ionomycin isolated from microalgae. Extraction of the crude microalgae aqueous extract and subsequent fractionation with solvents with increasing polarity concentrated the activity in more polar fractions.

Ischemic disorders involving platelet aggregation and blood coagulation are the leading cause of disability and death worldwide. Antithrombotic therapy is unsatisfactory and may cause side effects. Thus, there is a need to search for molecules with antithrombotic properties. Marine organisms produce substances with various well-defined environmental functions. In addition, some of these molecules also exhibit pharmacological properties, such as antiviral, anticancer, antiphoid and anticoagulant properties. The study aimed to evaluate, using in vitro tests, the effect of two brown algae extracts and ten marine sponges from Brazil on platelet aggregation and blood coagulation. The results showed that most extracts were able to inhibit platelet aggregation and clotting, as measured by plasma recalcification tests, prothrombin time, activated partial thromboplastin time and fibrinogenolytic activity. On the other hand, five out of ten species of sponges caused platelet aggregation. Marine organisms studied in this paper may have molecules with antithrombotic properties that represent the biotechnological potential for antithrombotic therapy. Further chemical studies should be conducted on active species in order to discover useful molecules for the development of new drugs for the treatment of coagulation disorders [120].

## 9. Conclusions

Microalgae are considered a valuable biological resource, and in recent years they have received great attention. Their economic importance worldwide is associated with a wide range of applications of microalgae, from the food industry to medicine, from immunostimulants to biofuels, from cosmetology to agriculture. They are able to synthesize proteins, polysaccharides, lipids, polyunsaturated fatty acids, vitamins, pigments, enzymes, phycobiliproteins, etc. Biologically active substances from microalgae exhibit antioxidant, antibacterial, antiviral, antitumor, regenerative, antihypertensive, neuroprotective and immunostimulating effects. Microalgae biomass is a promising source of both nutritional and functional additives.

Only a few species of microalgae (*Arthrospira (Spirulina) platensis, Chlorella* or *Chlorella vulgaris, Dunaliella, Aphanizomenon* and *Nostoc*) are allowed for human consumption. These microalgae are a promising object for large-scale cultivation due to the high content of biologically active substances and the relatively cheap manufacturing process. Other microalgae species such as *Chlamydomonas* sp., *Chlorococcum* sp., *Scenedescmus* sp., *Tetraselmis chuii and Nanochloropsis* sp. have established themselves as a source of useful components in aquaculture, feed, fertilizers and cosmetics, but they do not have GRAS (Generally recognized as safe) status yet [93].

Due to the wide variety of microalgae, high metabolic flexibility and various cultivation conditions, their real potential has not yet been fully evaluated. Researchers working with microalgae are facing the following tasks:

– improvement of photobioreactors;
– improvement of strain productivity (selection and genetic engineering methods);
– search for strains with new properties;
– study of the influence of cultivation conditions on the content of biologically active substances in cells;
– optimization of cultivation processes (lower costs and increased product yield);
– greening of production (introduction of closed production cycles, reducing waste, the most comprehensive use of useful components);
– assessment of environmental, economic and medical risks of scaling up production.

The multifaceted and joint work on these tasks will make it possible to replace the currently unstable production processes with alternative, less destructive ones. Innovative developments for the microalgae production optimization will make their use economically feasible and sought-after in the future.

## Figures and Tables

**Table 1:** The influence of temperature on the growth of various microalgae (table reconstructed using data from Varshney et al. [7]).

Microalgae	Biomass Yield, g/L		
	27 °C	30 °C	35 °C
*Chlorella vulgaris*	0.77	0.83	0.36
*Chlamydomonas reinhardtii*	0.33	0.75	0.18
*Botryococcus braunii*	0.79	0.81	0.64
*Scenedesmus obliquus*	0.65	0.67	0.23
*Neochloris cohaerens*	0.58	0.77	0.25
*Haematococcus pluvialis*	0.68	0.72	0.55
*Nannochloropsis gaditana*	0.45	0.59	0.13

**Table 2:** The influence of cultivation time on the growth of various microalgae (table reconstructed using data from Qiu et al. [8]).

Microalgae	Biomass Yield, g/L		
	7 days	14 days	21 days
*Chlorella vulgaris*	0.68	0.72	0.47
*Chlamydomonas reinhardtii*	0.78	0.79	0.65
*Botryococcus braunii*	0.64	0.69	0.37
*Scenedesmus obliquus*	0.75	0.80	0.29
*Neochloris cohaerens*	0.76	0.79	0.52
*Haematococcus pluvialis*	0.73	0.75	0.67
*Nannochloropsis gaditana*	0.68	0.73	0.53

**Table 3:** The influence of culture stirring on the growth of various microalgae (table reconstructed using data from Varshney et al. [7]).

Microalgae	Biomass Yield, g/L		
	0 rpm	50 rpm	100 rpm
*Chlorella vulgaris*	0.24	0.66	0.32
*Chlamydomonas reinhardtii*	0.39	0.62	0.42
*Botryococcus braunii*	0.26	0.59	0.33
*Scenedesmus obliquus*	0.43	0.69	0.52
*Neochloris cohaerens*	0.41	0.71	0.59
*Haematococcus pluvialis*	0.23	0.63	0.67
*Nannochloropsis gaditana*	0.36	0.70	0.55

**Table 4:** The influence of pH on the growth of various microalgae (table reconstructed using data from Varshney et al. [7]).

Microalgae	Biomass Yield, g/L		
	pH = 4	pH = 6	pH = 8
*Chlorella vulgaris*	0.75	0.67	0.34
*Chlamydomonas reinhardtii*	0.17	0.72	0.68
*Botryococcus braunii*	0.16	0.74	0.78
*Scenedesmus obliquus*	0.18	0.65	0.69
*Neochloris cohaerens*	0.21	0.73	0.68
*Haematococcus pluvialis*	0.16	0.69	0.67
*Nannochloropsis gaditana*	0.18	0.78	0.76

**Table 5:** Optimal conditions for growing microalgae biomass (table reconstructed using data from Nancucheo and Johnson [9]).

Microalgae	Cultivation Temperature, °C	Cultivation Duration, Days	Culture Stirring, rpm	pH of Culture Medium
*Chlorella vulgaris*	29	13	60	4.1
*Chlamydomonas reinhardtii*	30	13	75	6.3
*Botryococcus braunii*	27	11	70	6.8
*Scenedesmus obliquus*	29	12	90	7.1
*Neochloris cohaerens*	27	12	85	6.9
*Haematococcus pluvialis*	28	14	90	7.3
*Nannochloropsis gaditana*	30	11	70	7.6

**Table 6:** Characteristics of possible methods for microalgae cultivation.

Cultivation Method	Energy Source	Carbon Source	Cell Biomass Accumulation Rate	Reactor Type	Price	Features
Phototrophic	Light	Inorganic	Low	Photobioreactor/open waters	Low	The cell density of the culture is low; water evaporation
Heterotrophic	Organic matter	Organic	High	Bioreactor	Medium	The high price of the nutrient medium components; possibility of microbial contamination
Mixotrophic	Light, organic matter	Organic and inorganic	Medium	Closed photobioreactor	High	The high price of the nutrient medium components; possibility of microbial contamination

**Table 7:** Advantages and disadvantages of various methods for protein isolation from microalgae biomass (table built using combined data from previous studies [48,49,50,51,52,53,54,55,56,57,58]).

Protein Isolation Method	Advantages	Disadvantages
Electrochemical methods	applicable for all types of microalgae; no chemicals required	electrodes required; high costs of electricity and equipment
Flotation	widespread use in industry; low cost and space requirements; process time	surfactants are required; the high cost of ozone flotation
Coagulation/flocculation	quick and easy method; possible widespread use; less cellular damage; fewer energy costs; universal for most types of microalgae	the high cost of chemicals; high pH dependence; the difficulty of separating the coagulant from the biomass; dependence of effectiveness on the used coagulant; limited reuse of the medium; the possibility of bacterial or mineral contamination
Filtration	high efficiency; chemicals are not required; low energy consumption (pressure filtration and conventional); water recycling	not suitable for microalgae with a size of 3–30 microns; clogged membranes and filter replacement increase the cost of the process; high power consumption (vacuum filtration)
Centrifugation	high biomass collection efficiency (>90); preferable for small-scale enterprises and laboratories; suitable for collecting high-value biomass products	high cost and energy consumption; high maintenance cost

**Table 8:** The results of the study of the fatty acid composition of the microalgae lipid fraction (% of total lipids; table built using combined data from previous studies [60,61,62,63,64,65,66,67,68,69,70,71,72]).

Fatty Acids *	*Chlorella vulgaris*	*Botryococcus braunii*	*Neochloris cohaerens*	*Chlamydomonas reinhardtii*	*Nannochloropsis gaditana*
C14:0	1.15 ± 0.03	2.21 ± 0.06	0.72 ± 0.01	–	–
C14:1	−	–	–	2.38 ± 0.07	2.39 ± 0.07
C15:0	–	10.92 ± 0.34	0.13 ± 0.01	0.79 ± 0,02	1.05 ± 0.03
C16:0	13.65 ± 0.47	–	20.48 ± 0.61	17.25 ± 0.51	16.18 ± 0.48
C16:1	1.23 ± 0.03	5.04 ± 0.15	2.79 ± 0.08	–	1.71 ± 0.05
C16:2	1.84 ± 0.05	2.76 ± 0.08	–	3.13 ± 0.09	–
C16:3	–	4.93 ± 0.14	0.21 ± 0.01	–	37.87 ± 1.13
C17:0	2.19 ± 0.06	21.54 ± 0.70	0.15 ± 0.01	1.64 ± 0.04	4.34 ± 0.12
C17:1	–	–	–	21.28 ± 0.63	0.59 ± 0.01
C18:0	38.51 ± 1.21	–	42.97 ± 1.31	42.81 ± 1.28	–
C18:1	16.79 ± 0.55	14.68 ± 0.44	–	–	22.26 ± 0.66
C18:2	7.02 ± 0.20	–	8.03 ± 0.23	6.29 ± 0.21	8.27 ± 0.25
C18:3	1.47 ± 0.04	6.42 ± 0.12	3.06 ± 0.09	1.73 ± 0.04	3.78 ± 0.10
C20:0	1.22 ± 0.03	7.18 ± 0.21	4.44 ± 0.13	–	–
C22:0	–	4.29 ± 1.84	–	–	0.52 ± 0.01
C22:5	1.12 ± 0.03	–	0.38 ± 0.01	1.31 ± 0.03	1.49 ± 0.04
C22:6	7.87 ± 0.23	17.51 ± 0.51	–	–	–
C24:0	–	–	15.19 ± 0.48	–	0.56 ± 0.01
C24:1	–	–	0.23 ± 0.01	0.68 ± 0.02	–

* C14:0—myristic acid; C14:1—myristooleic acid; C15:0—pentadecanoic acid; C16:0—palmitic acid; C16:1—palmitoleic acid; C16:2—hexadecadienoic acid; C16:3—hexadecatrienic acid; C17:0—heptadecanoic acid; C17:1—cis-10-Heptadecenoic acid; C18:0—stearic acid; C18:1—oleic acid; C18:2—linoleic acid; C18:3—linolenic acid; C20:0—arachinic acid; C22:0—behenic acid; C22:5—docosapentaenoic acid; C22:6—docosahexaenoic acid; C24:0—lignoceric acid; C24:1—nervonic acid.

**Table 9:** Chlorophyll a and b content in microalgae (table built using combined data from previous studies [79,80,81,82,83,84,85,86,87,88,89,90,91]).

Microalgae	Content, %	
	Chlorophyll a	Chlorophyll b
*Chlorella vulgaris*	4.18 ± 0.12	2.53 ± 0.07
*Chlamydomonas reinhardtii*	3.48 ± 0.09	3.61 ± 0.10
*Botryococcus braunii*	3.72 ± 0.09	2.17 ± 0.07
*Scenedesmus obliquus*	3.56 ± 0.09	6.58 ± 0.19
*Neochloris cohaerens*	6.13 ± 0.18	1.57 ± 0.05
*Haematococcus pluvialis*	3.84 ± 0.13	2.46 ± 0.90
*Nannochloropsis* *gaditana*	3.71 ± 0.10	1.72 ± 0.04

## References

[1] Olasehinde T.A., Olaniran A.O., Okoh A.I. (2017). Therapeutic potentials of microalgae in the treatment of Alzheimer’s disease. Molecules.

[2] Demirel Z., Yılmaz F.F., Ozdemir G., Dalay M.C. (2018). Influence of media and temperature on the growth and the biological activities of Desmodesmus protuberans (F.E. Fritsch & M.F. Rich) E. Hegewald. Turk. J. Fish. Aquat. Sci..

[3] Gürlek C., Yarkent C., Köse A., Oral I., Öncel S.S., Elibol M. (2019). Evaluation of several microalgal extracts as bioactive metabolites as potential pharmaceutical compounds. CMBEBIH 2019.

[4] Bhattacharjee M. (2016). Pharmaceutically valuable bioactive compounds of algae. Asian J. Pharm. Clin. Res..

[5] Villarruel-Lopez A., Ascencio F., Nuno K. (2017). Microalgae, a potential natural functional food source—A review. Pol. J. Food Nutr. Sci..

[6] Singh S.P., Singh P. (2015). Effect of temperature and light on the growth of algae species: A review. Renew. Sustain. Energy Rev..

[7] Varshney P., Beardall J., Bhattacharya S., Wangikar P.P. (2018). Isolation and biochemical characterisation of two thermophilic green algal species—*Asterarcys quadricellulare* and *Chlorella sorokiniana*, which are tolerant to high levels of carbon dioxide and nitric oxide. Algal Res..

[8] Qiu R., Gao S., Lopez P.A., Ogden K.L. (2017). Effects of pH on cell growth, lipid production and CO_2_ addition of microalgae Chlorella sorokiniana. Algal Res..

[9] Nancucheo I., Johnson D.B. (2012). Acidophilic algae isolated from mine-impacted environments and their roles in sustaining heterotrophic acidophiles. Front. Microbiol..

[10] Valdés F.J., Hernández M.R., Catalá L., Gomis A.M. (2012). Estimation of CO_2_ stripping/CO_2_ microalgae consumption ratios in a bubble column photobioreactor using the analysis of the pH profiles. Application to nannochloropsis oculata microalgae culture. Bioresour. Technol..

[11] Mohan S.V., Devi M.P. (2014). Salinity stress induced lipid synthesis to harness biodiesel during dual mode cultivation of mixotrophic microalgae. Bioresour. Technol..

[12] De Alva M.S., Pabello V.M.L., Ledesma M.T.O., Gómez M.J.C. (2018). Carbon, nitrogen, and phosphorus removal, and lipid production by three saline microalgae grown in synthetic wastewater irradiated with different photon fluxes. Algal Res..

[13] Markou G., Vandamme D., Muylaert K. (2014). Microalgal and cyanobacterial cultivation: The supply of nutrients. Water Res..

[14] Kadkhodeaei S., Abbasiliasi S., Shun T.J., Masoumi H.R.F., Mohamed M.S., Movahedi A., Rahim R., Ariff A.B. (2015). Enhancement of protein production by microalgae *Dunaliella salina* under mixotrophic condition using response surface methodology. RSC Adv..

[15] Camacho-Rodríguez J., Cerón-García M.C., Fernández-Sevilla J.M., Molina-Grima E. (2015). The influence of culture conditions on biomass and high value product generation by Nannochloropsis gaditana in aquaculture. Algal Res..

[16] Goiris K., Van-Colen W., Wilches I., León-Tamariz F., De Cooman L., Muylaert K. (2015). Impact of nutrient stress on antioxidant production in three species of microalgae. Algal Res..

[17] Nurachman Z., Hartini H., Rahmaniyah W.R., Kurnia D., Hidayat R., Prijamboedi B., Suendo V., Ratnaningsih E., Panggabean L.M.G., Nurbaiti S. (2015). Tropical marine *Chlorella* sp. PP1 as a source of photosynthetic pigments for dye-sensitized solar cells. Algal Res..

[18] Durán I., Rubiera F., Pevida C. (2018). Microalgae: Potential precursors of CO_2_ adsorbents. J. CO2 Util..

[19] Pires J.C.M., Alvim-Ferraz M.C.M., Martins F.G., Simões M. (2012). Carbon dioxide capture from flue gases using microalgae: Engineering aspects and biorefinery concept. Renew. Sustain. Energy Rev..

[20] Iasimone F., Panico A., Felice V., Fantasma F., Iorizzi M., Pirozzi F. (2018). Effect of light intensity and nutrient supply on microalgae cultivated in urban wastewater: Biomass production, lipids accumulation and settleability characteristics. J. Environ. Manag..

[21] Corrêa D.O., Santos B., Dias F.G., Vargas J.V.C., Mariano A.B., Balmant W., Rosa M.P., Savi D.C., Kava V., Glienke C. (2017). Enhanced biohydrogen production from microalgae by diesel engine hazardous emissions fixation. Int. J. Hydrog. Energy.

[22] Tolfo da Fontoura J., Rolim G.S., Farenzena M., Gutterres M. (2017). Influence of light intensity and tannery wastewater concentration on biomass production and nutrient removal by microalgae *Scenedesmus* sp.. Process. Saf. Environ. Prot..

[23] Rai M.P., Gupta S. (2017). Effect of media composition and light supply on biomass, lipid content and fame profile for quality biofuel production from Scenedesmus abundans. Energy Convers. Manag..

[24] Nikolaou A., Hartmann P., Sciandra A., Chachuat B., Bernard O. (2016). Dynamic coupling of photoacclimation and photoinhibition in a model of microalgae growth. J. Theor. Biol..

[25] Zhang X., Tang X., Zhou B., Hu S., Wang Y. (2015). Effect of enhanced UV-B radiation on photosynthetic characteristics of marine microalgae Dunaliella Salina (Chlorophyta, Chlorophyceae). J. Exp. Mar. Biol. Ecol..

[26] Fu W., Guomundsson O., Paglia G., Herjolfsson G., Andrésson O.S., Palsson B.O., Brynjolfsson S. (2013). Enhancement of carotenoid biosynthesis in the green microalga Dunaliella Salina with light-emitting diodes and adaptive laboratory evolution. Appl. Microbiol. Biotechnol..

[27] Takache H., Pruvost J., Marec H. (2015). Investigation of light/dark cycles effects on the photosynthetic growth of Chlamydomonas Reinhardtii in conditions representative of photobioreactor cultivation. Algal Res..

[28] Chew K.W., Chia S.R., Show P.L., Yap Y.J., Ling T.C., Chang J.S. (2018). Effects of water culture medium, cultivation systems and growth modes for microalgae cultivation: A review. J. Taiwan Inst. Chem. Eng..

[29] Trenkenshu R.P. (2016). Dinamicheskaya model biotransformacii zapovednoy i strukturnoy biomassy formy mikrovodorosley v temnote (The dynamic model of biotransformation of reserved and structural biomass form of microalgae in darkness). Voprosy sovremennoy geologii (Quest. Mod. Algol.).

[30] Zhu L. (2015). Microalgal culture strategies for biofuel production: A review. Biofuels Bioprod. Biorefin..

[31] Wang C., Lan C.Q. (2018). Effects of shear stress on microalgae: A review. Biotechnol. Adv..

[32] Anyanwu R.C., Rodriguez C., Durrant A., Olabi A.G. (2018). Microalgae cultivation technologies. Reference Module in Materials Science and Materials Engineering.

[33] Sánchez A., Maceiras R., Cancela A., Pérez A. (2013). Culture aspects of Isochrysis galbana for biodiesel production. Appl. Energy.

[34] Hu J., Nagarajan D., Zhang Q., Chang J.S., Lee D.J. (2018). Heterotrophic cultivation of microalgae for pigment production: A review. Biotechnol. Adv..

[35] Zhan J., Rong J., Wang Q. (2017). Mixotrophic cultivation, a preferable microalgae cultivation mode for biomass/bioenergy production, and bioremediation, advances and prospect. Int. J. Hydrog. Energy.

[36] Rincon S.M., Urrego N.F., Avila K.J., Romero H.M., Beyenal H. (2019). Photosynthetic activity assessment in mixotrophically cultured Chlorella vulgaris biofilms at various developmental stages. Algal Res..

[37] Cassini S.T., Francisco S.A., Antunes P.W.P., Nunes Oss R., Keller R. (2017). Harvesting microalgal biomass grown in anaerobic sewage treatment effluent by the coagulation-flocculation method: Effect of pH. Braz. Arch. Biol. Technol..

[38] Venkata Mohan S., Rohit M.V., Chiranjeevi P., Chandra R., Navaneeth B. (2015). Heterotrophic microalgae cultivation to synergize biodiesel production with waste remediation: Progress and perspectives. Bioresour. Technol..

[39] Zittelli G.C., Biondi N., Rodolfi L., Tredici M.R., Richmond A., Hu Q. (2013). Photobioreactors for mass production of microalgae. Handbook of Microalgal Culture: Applied Phycology and Biotechnology.

[40] Lafarga T. (2019). Cultured microalgae and compounds derived thereof for food applications: Strain selection and cultivation, drying, and processing strategies. Food Rev. Int..

[41] Deruyck B., Thi Nguyen K.H., Decaestecker E., Muylaert K. (2019). Modeling the impact of rotifer contamination on microalgal production in open pond, photobioreactor and thin layer cultivation systems. Algal Res..

[42] Kannan D.C., Venkat D. (2019). An open outdoor algal growth system of improved productivity for biofuel production. J. Chem. Technol. Biotechnol..

[43] Pruvost J., Le Gouic B., Lepine O., Legrand J., Le Borgne F. (2016). Microalgae culture in building-integrated photobioreactors: Biomass production modelling and energetic analysis. Chem. Eng. J..

[44] Morales-Sánchez D., Martinez-Rodriguez O.A., Martinez A. (2016). Heterotrophic cultivation of microalgae: Production of metabolites of commercial interest. J. Chem. Technol. Biotechnol..

[45] Wang B., Lan C.Q., Horsman M. (2012). Closed photobioreactors for production of microalgal biomasses. Biotechnol. Adv..

[46] Venkata Mohan S., Hemalatha M., Chakraborty D., Chatterjee S., Ranadheer P., Kona R. (2019). Algal biorefinery models with self-sustainable closed loop approach: Trends and prospective for blue-bioeconomy. Bioresour. Technol..

[47] Ejike C.E.C.C., Collins S.A., Balasuriya N., Swanson A.K., Mason B., Udenigwe C.C. (2017). Prospects of microalgae proteins in producing peptide-based functional foods for promoting cardiovascular health. Trends Food Sci. Technol..

[48] Safi C., Ursu A.V., Laroche C., Zebiba B., Merah O., Pontalier P.Y., Vaca-Garcia C. (2014). Aqueous extraction of proteins from microalgae: Effect of different cell disruption methods. Algal Res..

[49] Barka A., Blecker C. (2016). Microalge as a potential source of singelcell proteins: A review. Biotechnol. Agron. Soc. Environ..

[50] Templeton D.W., Laurens L.M.L. (2015). Nitrogen-to-protein conversion factors revisited for application of microalga biomass conversion to food, feed and fuel. Algal Res..

[51] Lim A.S., Jeong H.J., Kim S.J., Hee Ok J. (2018). Amino acids profiles of six dinoflagellate species belonging to diverse families: Possible use as animal feeds in aquaculture. Algae.

[52] Vernèsa L., Abert-Viana M., El Maâtaouib M., Tao Y., Bornard I., Chemat F. (2019). Application of ultrasound for green extraction of proteins from spirulina. Mechanism, optimization, modeling, and industrial prospects. Ultrason. Sonochem..

[53] Xie T., Xia Y., Zeng Y., Li X., Zhang Y. (2017). Nitrate concentration-shift cultivation to enhance protein content of heterotrophic microalga Chlorella vulgaris: Over-compensation strategy. Bioresour. Technol..

[54] Waghmare A.G., Salve M.K., LeBlanc J.G., Arya S.S. (2016). Concentration and characterization of microalgae proteins from Chlorella pyrenoidosa. Bioresour. Bioprocess..

[55] Chia S.R., Chew K.W., Zaid H.F.M., Chu D.T., Tao Y., Show P.L. (2019). Microalgal protein extraction from Chlorella vulgaris FSP-E using triphasic partitioning technique with sonication. Front. Bioeng. Biotechnol..

[56] Lupatini A.L., Bispo L.O., Colla L.M., Vieira Costa J.A., Canan C., Colla E. (2017). Protein and carbohydrate extraction from S. platensis biomass by ultrasound and mechanical agitation. Food Res. Int..

[57] Safi C., Olivieri G., Campos R.P., Engelen-Smit N., Mulder W.J., van den Broek L.A.M., Sijtsma L. (2017). Biorefinery of microalgal soluble proteins by sequential processing and membrane filtration. Bioresour. Technol..

[58] Kose A., Ozen M.O., Elibol M., Oncel S.S. (2017). Investigation of in vitro digestibility of dietary microalga Chlorella vulgaris and cyanobacterium Spirulina platensis as a nutritional supplement. 3 Biotech.

[59] Nee W., Le C.F., Show P.L., Lam H.L., Ling T.C. (2016). Evaluation of different solvent types on the extraction of proteins from microalgae. Chem. Eng. Trans..

[60] Bellou S., Baeshen M., Elazzazy A.M., Aggeli D., Sayegh F., Aggelis G. (2014). Microalgal lipids biochemistry and biotechnological perspectives. Biotechnol. Adv..

[61] Sprague M., Betancor M.B., Tocher D.R. (2017). Microbial and genetically engineered oils as replacements for fish oil in aquaculture feeds. Biotechnol. Lett..

[62] Ferreira G.F., Ríos Pinto L.F., Maciel Filho R., Fregolente L.V. (2019). A review on lipid production from microalgae: Association between cultivation using waste streams and fatty acid profiles. Renew. Sustain. Energy Rev..

[63] Scharff C., Domurath N., Wensch-Dorendorf M., Schröder F.G. (2017). Effect of different photoperiods on the biochemical profile of the green algae *C. vulgaris* and *S. obliquus*. Acta Hortic..

[64] Chandra T.S., Aditi S., Kumar M.M., Mukherji S., Modak J., Chauhan V.S., Sarada R., Mudliar S.N. (2017). Growth and biochemical characteristics of an indigenous freshwater microalga, Scenedesmus obtusus, cultivated in an airlift photobioreactor: Effect of reactor hydrodynamics, light intensity, and photoperiod. Bioprocess. Biosyst. Eng..

[65] Choi Y.K., Kim H.J., Kumaran R.S., Song H.J., Song K.G., Kim K.J., Lee S.H., Yang Y.H., Kim H.J. (2017). Enhanced growth and total fatty acid production of microalgae under various lighting conditions induced by flashing light. Eng. Life Sci..

[66] Carpio R.B., De Leon R.L., Martinez-Goss M.R. (2015). Growth, lipid content, and lipid profile of the green alga, Chlorella vulgaris beij., under different concentrations of Fe and CO_2_. J. Eng. Sci. Technol..

[67] Liu J.X., Tan K.T., He L.J., Qiu Y.T., Tan W.L., Guo Y., Wang Z.H., Sun W. (2018). Effect of limitation of iron and manganese on microalgae growth in fresh water. Microbiology.

[68] Abd El Baky H.H., El-Baroty G.S., Bouaid A. (2014). Lipid induction in *Dunaliella salina* culture aerated with various levels CO_2_ and its biodiesel production. J. Aquac. Res. Dev..

[69] Ramirez-Lopez C., Chairez I., Fernandez-Linares L. (2016). A novel culture medium designed for the simultaneous enhancement of biomass and lipid production by Chlorella vulgaris UTEX 26. Bioresour. Technol..

[70] Josephine A., Niveditha C., Radhika A., Shali A.B., Kumar T.S., Dharani G., Kirubagaran R. (2015). Analytical evaluation of different carbon sources and growth stimulators on the biomass and lipid production of *Chlorella vulgaris*—Implications for biofuels. Biomass Bioenergy.

[71] Lukashiv O.Y., Grubinko V.V. (2017). The influence of a selenium-chromium-lipid complex obtained from Chlorella vulgaris on the energy metabolism in rats with experimental diabetes. Regul. Mech. Biosyst..

[72] Anthony J., Sivashankarasubbiah K.T., Thonthula S., Rangamaran V.R., Gopal D., Ramalingam K. (2018). An efficient method for the sequential production of lipid and carotenoids from the Chlorella Growth Factor-extracted biomass of Chlorella vulgaris. J. Appl. Phycol..

[73] Chou N.-T., Cheng C.-F., Wu H.-C., Lai C.-P., Lin L.-T., Pan I.-H., Ko C.-H. (2012). Chlorella sorokiniana-Induced activation and maturation of human monocyte-derived dendritic cells through NF-κB and PI3K/MAPK pathways. Evid. Based Complement. Alternat. Med..

[74] Song H., He M., Gu C., Wei D., Liang Y., Yan J., Wang C.H. (2018). Extraction optimization, purification, antioxidant activity, and preliminary structural characterization of crude polysaccharide from an arctic *Chlorella* sp.. Polymers.

[75] Liu Q.S., Yao C.H., Sun Y.X., Chen W., Tan H.D., Cao X.P., Xue S., Yin H. (2019). Production and structural characterization of a new type of polysaccharide from nitrogen-limited Arthrospira platensis cultivated in outdoor industrial-scale open raceway ponds. Biotechnol. Biofuels.

[76] El-Ahmady El-Naggar N., Hussein M.H., Shaaban-Dessuuki S.A., Dalal S.R. (2020). Production, extraction and characterization of Chlorella vulgaris soluble polysaccharides and their applications in AgNPs biosynthesis and biostimulation of plant growth. Sci. Rep..

[77] Liu X., Zhang M., Liu H., Zhou A., Cao Y., Liu X. (2018). Preliminary characterization of the structure and immunostimulatory and anti-aging properties of the polysaccharide fraction of *Haematococcus pluvialis*. RSC Adv..

[78] Gaignard C., Laroche C., Pierre G., Dubessay P., Delattre C., Gardarin C., Gourvil P., Probert I., Dubuffet A., Michaud P. (2019). Screening of marine microalgae: Investigation of new exopolysaccharide producers. Algal Res..

[79] Koller M., Muhr A., Braunegg G. (2014). Microalgae as versatile cellular factories for valued products. Algal Res..

[80] Mulders K.J.M., Weesepoel Y., Lamers P.P., Vincken J.P., Martens D.E., Wijffels R.H. (2013). Growth and pigment accumulation in nutrient-depleted Isochrysis galbana T-ISO. J. Appl. Phycol..

[81] D’Alessandro E.B., Antoniosi Filho N.R. (2016). Concepts and studies on lipid and pigments of microalgae: A review. Renew. Sustain. Energy Rev..

[82] Cuellar-Bermudez S.P., Aguilar-Hernandez I., Cardenas-Chavez D.L., Ornelas-Soto N., Romero-Ogawa M.A., Parra-Saldivar R. (2015). Extraction and purification of high-value metabolites from microalgae: Essential lipids, astaxanthin and phycobiliproteins. Microb. Biotechnol..

[83] Kumudha A., Selvakumar S., Dilshad P., Vaidyanathan G., Thakur M.S., Sarada R. (2015). Methylcobalamin—A form of vitamin B12 identified and characterised in Chlorella vulgaris. Food Chem..

[84] Santiago-Morales I.S., Trujillo-Valle L., Márquez-Rocha F.J., López Hernández J.F. (2018). Tocopherols, Phycocyanin and Superoxide Dismutase from Microalgae as Potential Food Antioxidants. Appl. Food Biotechnol..

[85] Smerilli A., Orefice I., Corato F., Ruban A., Brunet C. (2017). Photoprotective and antioxidant responses to light spectrum and intensity variations in the coastal diatom Skeletonema marinoi. Environ. Microbiol..

[86] Papadaki S., Kyriakopoulou K., Magdalini K. (2017). Recovery and Encapsualtion of Bioactive Extracts from Haematococcus pluvialis and Phaedodactylum tricornutum for food Applications. IOSR J. Environ. Sci. Toxicol. Food Technol..

[87] Grudzinski W., Krzeminska I., Luchowski R., Nosalewicz A., Gruszecki W.I. (2016). Strong-light-induced yellowing of green microalgae Chlorella: A study on molecular mechanisms of the acclimation response. Algal Res..

[88] Soares A.T., Marques Júnior J.G., Lopes R.G., Derner R.B., Antoniosi Filho N.R. (2016). Improvement of the extraction process for high commercial value pigments from *Desmodesmus* sp. microalgae. J. Braz. Chem. Soc..

[89] Pataro G., Carullo D., Ferrari G. (2019). PEF-assisted supercritical CO_2_ extraction of pigments from microalgae Nannochloropsis oceanica in a continuous flow system. Chem. Eng. Trans..

[90] Singh P., Baranwal M., Reddy S.M. (2016). Antioxidant and cytotoxic activity of carotenes produced by *Dunaliella salina* under stress. Pharm. Biol..

[91] Sathasivam R., Pongpadung P., Praiboon J., Chirapart A., Trakulnaleamsai S., Roytrakul S., Juntawong N. (2018). Optimizing NaCl and KNO_3_ concentrations for high β-carotene production in photobioreactor by *Dunaliella salina* KU11 isolated from saline soil sample. Chiang Mai J. Sci..

[92] Bray F., Ferlay J., Soerjomataram I., Siegel R.L., Torre L.A., Jemal A. (2018). Global cancer statistics 2018: GLOBOCAN estimates of incidence and mortality worldwide for 36 cancers in 185 countries. CA Cancer J. Clin..

[93] Rizwan M., Mujtaba G., Memon S.A., Lee K., Rashid N. (2018). Exploring the potential of microalgae for new biotechnology applications and beyond: A review. Renew. Sustain. Energy Rev..

[94] Galasso C., Gentile A., Orefice I., Ianora A., Bruno A., Noonan D.M., Sansone C., Albini A., Brunet C. (2019). Microalgal Derivatives as potential nutraceutical and food supplements for human health: A focus on cancer prevention and interception. Nutrients.

[95] Lauritano C., Andersen J.H., Hansen E., Albrigtsen M., Escalera L., Esposito F., Helland K., Hanssen K.O., Romano G., Ianora A. (2016). Bioactivity screening of microalgae for antioxidant, anti-inflammatory, anticancer, anti-diabetes, and antibacterial activities. Front. Mar. Sci..

[96] Somasekharan S.P., El-Naggar A., Sorensen P.H., Wang Y., Cheng H. (2016). An aqueous extract of marine microalgae exhibits antimetastatic activity through preferential killing of suspended cancer cells and anticolony forming activity. Evid. Based Complement. Alternat. Med..

[97] Chen X.L., Song L., Wang H., Liu S., Yu H.H., Wang X.Q., Li R.F., Liu T.Z., Li P.C. (2019). Partial characterization, the immune modulation and anticancer activities of sulfated polysaccharides from filamentous microalgae *Tribonema* sp.. Molecules.

[98] Samarakoon K.W., Ko J.Y., Lee J.H., Kwon O.N., Kim S.W., Jeon Y.J. (2014). Apoptotic anticancer activity of a novel fatty alcohol ester isolated from cultured marine diatom, *Phaeodactylum tricornutum*. J. Funct. Foods.

[99] Jabeen A., Reeder B.J., Hisaindee S., Ashraf S., Al Darmaki N., Battah S., Al-Zuhair S. (2017). Effect of Enzymatic pre-treatment of microalgae extracts on their anti-tumor activity. Biomed. J..

[100] Falaise C., François C., Travers M.A., Morga B., Haure J., Tremblay R., Turcotte F., Pasetto P., Gastineau R., Hardivillier Y. (2016). Antimicrobial Compounds from eukaryotic microalgae against human pathogens and diseases in aquaculture. Mar. Drugs.

[101] Krishnakumar S., Bai V.D.M., Rajan R.A. (2013). Evaluation of bioactive metabolites from halophilic microalgae Dunaliella Salina by GC—MS analysis. Int. J. Pharm. Pharm. Sci..

[102] Kilic N.K., Erdem K., Donmez G. (2018). Bioactive compounds produced by Dunaliella species, antimicrobial effects and optimization of the efficiency. Turk. J. Fish. Aquat. Sci..

[103] Tessmer Scaglioni P., Quadros L., de Paula M., Badiale Furlong V., César Abreu P., Badiale-Furlong E. (2018). Inhibition of enzymatic and oxidative processes by phenolic extracts from *Spirulina* sp. and *Nannochloropsis* sp.. Food Technol. Biotechnol..

[104] Patel A., Matsakas L., Hruzová K., Rova U., Christakopoulos P. (2019). Biosynthesis of nutraceutical fatty acids by the oleaginous marine microalgae Phaeodactylum tricornutum utilizing hydrolysates from organosolv-pretreated birch and spruce biomass. Mar. Drugs.

[105] Joshi S., Kumari R., Upasani V.N. (2018). Applications of algae in cosmetics: An overview. Int. J. Innov. Sci. Eng. Technol..

[106] Sathasivam R., Ki J.S. (2018). A review of the biological activities of microalgal carotenoids and their potential use in healthcare and cosmetic industries. Mar. Drugs.

[107] Widowati I., Zainuri M., Kusumaningrum H.P., Susilowati R., Hardivillier Y., Leignel V., Bourgougnon N., Mouget J.-L. (2017). Antioxidant activity of three microalgae *Dunaliella salina*, Tetraselmis chuii and Isochrysis galbana clone Tahiti. IOP Conf. Ser. Earth Environ. Sci..

[108] Blagojević D., Babić O., Rašeta M., Šibul F., Janjušević L., Simeunović J. (2018). Antioxidant activity and phenolic profile in filamentous cyanobacteria: The impact of nitrogen. J. Appl. Phycol..

[109] Agregán R., Munekata P., Franco D., Carballo J., Barba F.J., Lorenzo J.M. (2018). Antioxidant potential of extracts obtained from macro-(*Ascophyllum nodosum*, *Fucus vesiculosus* and *Bifurcaria bifurcata*) and micro-algae (*Chlorella vulgaris* and *Spirulina platensis*) assisted by ultrasound. Medicines.

[110] Dantas D.M.d.M., Oliveira Y.B.d.C., Costa R.M.P.B., Carneiro-da-Cunha M.D.G., Gálvez A.O., Bezerra R.S. (2019). Evaluation of antioxidant and antibacterial capacity of green microalgae Scenedesmus subspicatus. Food Sci. Technol. Int..

[111] Sansone C., Galasso C., Orefice I., Nuzzo G., Luongo E., Cutignano A., Romano G., Brunet C., Fontana A., Esposito F. (2017). The green microalga Tetraselmis suecica reduces oxidative stress and induces repairing mechanisms in human cells. Sci. Rep..

[112] Hu X., Yang X.Q., Li L.H., Wu Y.Y., Lin W.L., Huang H., Yang S.L. (2015). Antioxidant properties of microalgae protein hydrolysates prepared by neutral protease digestion. Appl. Mech. Mater..

[113] Chen M.-F., Zhang Y.Y., He M.D., Li C.Y., Zhou C.X., Hong P.Z., Qian Z.-J. (2019). Antioxidant peptide purified from enzymatic hydrolysates of Isochrysis zhanjiangensis and its protective effect against ethanol induced oxidative stress of HepG2 Cells. Biotechnol. Bioprocess Eng..

[114] Chen L., Deng H., Cui H., Fang J., Zuo Z., Deng J., Li Y., Wang X., Zhao L. (2018). Inflammatory responses and inflammation-associated diseases in organs. Oncotarget.

[115] Khanra S., Mondal M., Halder G., Tiwari O.N., Gayen K., Bhowmick T.K. (2018). Downstream processing of microalgae for pigments, protein and carbohydrate in industrial application: A review. Food Bioprod. Process..

[116] Andrade L., Andrade C., Dias M., Nascimento C.A.O., Mendes M.A. (2018). Chlorella and Spirulina microalgae as sources of functional foods, nutraceuticals, and food supplements; an overview. MOJ Food Process. Technol..

[117] Raposo M.F., Morais A.M.M.B., Morais R.M.S.C. (2016). Emergent sources of prebiotics: Seaweeds and microalgae. Mar. Drugs.

[118] Raposo M.F., Morais R.M.S.C., Morai A.M.M.B. (2013). Bioactivity and applications of sulphated polysaccharides from marine microalgae. Mar. Drugs.

[119] Villar R., Martínez D., Núñez L., Jiménez C. (1997). Anti-aggregant effects on human platelets of the crude aqueous extract and polar fractions of the microalga *Dunaliella tertiolecta*. Phytother. Res..

[120] Moura L.D.A., Ortiz-Ramirez F., Cavalcanti D.N., Ribeiro S.M., Muricy G., Teixeira V.L., Fuly A.L. (2011). Evaluation of Marine Brown Algae and Sponges from Brazil as Anticoagulant and Antiplatelet Products. Mar. Drugs.

